# Systematic safety evaluation on photoluminescent carbon dots

**DOI:** 10.1186/1556-276X-8-122

**Published:** 2013-03-08

**Authors:** Kan Wang, Zhongcai Gao, Guo Gao, Yan Wo, Yuxia Wang, Guangxia Shen, Daxiang Cui

**Affiliations:** 1 National Key Laboratory of Nano/Micro Fabrication Technology, Institute of Micro-Nano Science and Technology, Shanghai Jiao Tong University, 800 Dongchuan Road, Shanghai, 200240, People’s Republic of China; 2 Institute of Pharmacology and Toxicology, Academy of Military Medical Sciences, Beijing, 100850, People’s Republic of China

**Keywords:** Carbon dots, Biocompatibility, Toxicity

## Abstract

Photoluminescent carbon dots (C-dots) were prepared using the improved nitric acid oxidation method. The C-dots were characterized by tapping-mode atomic force microscopy, and UV–vis absorption spectroscopy. The C-dots were subjected to systematic safety evaluation via acute toxicity, subacute toxicity, and genotoxicity experiments (including mouse bone marrow micronuclear test and *Salmonella typhimurium* mutagenicity test). The results showed that the C-dots were successfully prepared with good stability, high dispersibility, and water solubility. At all studied C-dot dosages, no significant toxic effect, i.e., no abnormality or lesion, was observed in the organs of the animals. Therefore, the C-dots are non-toxic to mice under any dose and have potential use in fluorescence imaging *in vivo*, tumor cell tracking, and others.

## Background

The importance of fluorescent nanoprobes in biomedical research and practice is rapidly increasing with the rapid developments in fluorescence microscopy, laser technologies, and nanotechnology. Fluorescent carbon dots (C-dots), a novel form of nanocarbon, have the inherent properties of traditional semiconductor-based quantum dots (e.g., size- and wavelength-dependent luminescence emissions, resistance to photobleaching, and ease of bioconjugation). Apart from these properties, C-dots also possess special features such as physicochemical stability, photochemical stability, and non-blinking behavior [1-3]. The preparation methods of C-dots are relatively simple, low cost, and applicable in large scales. Numerous approaches for synthesizing C-dots have been proposed, including dry methods (arc discharge [4,5] and laser ablation [6]) and solution methods (combustion/thermal [7-9], electrochemical oxidation [10], organic synthesis [11], and microwave methods [12-14]). C-dots are also potential replacements for conventional cadmium-based quantum dots used in biological imaging *in vitro* and *in vivo*[6,15-22] and have attracted significant attention since the pioneering work of Sun et al. [20,21].

Although carbon is not considered as an intrinsically toxic element, the specific material configurations and structures of C-dots may be potential risks to human health, thereby raising public concern [22]. Many toxicity evaluations have been conducted for various carbon nanomaterials in recent years, and the results of the different methods are discrepant [23-34]. The current work aimed to study systematically the toxicity of C-dot solution exposure in rats and mice by biochemical and hematological analyses. C-dots are found to have the advantages of chemical inertness, low cytotoxicity, and good biocompatibility.

## Main text

### Materials and methods

#### Preparation and characterization of carbon nanodots

C-dots were prepared using the improved nitric acid oxidation method. In a typical experiment, 0.5 g of raw soot (purchased from Jixi Kaiwen Hu, Co., Ltd., Jixi, China) was placed in acetone solution, ultrasonically cleaned for 30 min, centrifuged to discard the upper yellow solution, and then dried under a vacuum at 80°C. Subsequently, the cleaned soot was refluxed in 25 mL of 5 M HNO_3_ at 120°C for 12 to 18 h until a homogeneous black aqueous suspension was obtained. This black suspension was centrifuged at 3,000 rpm for 10 min to remove unreacted precipitates. The light-brown solution was collected, neutralized, and extensively dialyzed with an MWCO-1000 membrane against pure water. The suspended solution was precipitated by adding acetone and centrifuged at 14,000 rpm for 10 min. Size separation was performed in a water/ethanol/chloroform solvent mixture by high-speed (8,000 to 10,000 rpm) stepwise centrifugation. The supernatant was collected after spinning at 10,000 rpm, and the precipitate was discarded. Finally, a yellow solution of C-dots with 1- to 3-nm particle sizes was obtained. The C-dots were passivated with a PEG2000N solution at 140°C under the protection of nitrogen gas for 72 h. The dots were then dialyzed using an MCO 3000 dialysis membrane to remove excess PEG2000N.

Tapping-mode (TM)-atomic force microscopy (AFM) images of the C-dots -NH2 were taken using a MultiMode Nanoscope IIIa scanning probe microscopy system (Veeco Instruments Inc., Plainview, NY, USA). Commercially available AFM cantilever tips with a force constant of approximately 48 N/m and a resonance vibration frequency of approximately 330 kHz were used. The scanning rate was set to 1 to 1.5 Hz. The samples for TM-AFM were prepared by dropping an aqueous suspension (0.01 mg/mL) of C-dots NH2 on a freshly cleaved mica surface and drying under a vacuum at 80°C. UV–vis spectra were obtained at 20°C using a Shimadzu UV-2450 UV–vis spectrophotometer (Shimadzu Corporation, Kyoto, Japan) equipped with a 10-mm quartz cell and with a light path length of 1 cm. Fluorescence spectra were obtained using a Hitachi FL-4600 spectrofluorimeter (Hitachi Ltd., Tokyo, Japan).

#### Experimental animals

Systematic safety evaluation experiments (acute toxicity, subacute toxicity, and genotoxicity experiments, including mouse bone marrow micronuclear test and *Salmonella typhimurium* mutagenicity (Ames) test) were conducted. All animal experiments were performed in compliance with the local ethics committee. Animals were obtained from the animal laboratories of the Academy of Military Medical Sciences in compliance with the institutional Animal Care and Use Program Guidelines. The animals were given food and water, and housed under 12 h/12 h light/dark cycle. After acclimation, the animals were randomly divided into different groups for *in vivo* toxicity evaluations.

#### Acute toxicity evaluations

Sixty BALB/c mice (17 to 21 g) were divided into three groups of 20 each for tail injections to test for acute toxicity. Each group had 10 female and 10 male mice that were intravenously exposed to C-dots through a single tail injection of either 5.1 or 51 mg/kg body weight (BW). Other mice were injected with 0.9% NaCl aqueous solution to serve as the control group. Within 14 days of monitoring, the body weights of the mice were measured. At various time points (3 and 14 days after exposure), 10 mice (5 males and 5 females) per time point were sacrificed. Blood samples were collected from each mouse for blood chemistry tests and complete blood panel analysis. Statistical calculations were based on the standard deviations of 10 mice per group.

#### Subacute toxicity evaluations

Sixty-four Wistar rats (177 to 224 g) were randomly divided into three test groups (low, medium, or high dose) and one control group with 16 rats in each group (8 males and 8 females). The low, medium, and high doses (0.2, 2, and 20 mg/kg BW) of C-dots were administered as a single tail vein injection. The rats in the control group were exposed to 0.9% NaCl aqueous solution. At 1, 3, 7, and 28 days after exposure, blood from each rat was collected for blood chemistry tests and complete blood panel analyses before the rats were euthanatized 30 days post-exposure. The major organs (heart, liver, spleen, stomach, kidneys, lungs, brain, testicles, ovaries, adrenal glands, and intestines) were collected. For conventional histological analyses, tissues were immediately collected after the rats were sacrificed, fixed in 10% formaldehyde, embedded in paraffin, cut into 8-μm sections, stained with hematoxylin and eosin, and then examined by light microscopy. The results are presented as the mean ± SD. Statistical differences were evaluated using the variance test and considered significant at *P* < 0.05.

#### Medullary micronucleus test

Fifty healthy Kunming mice (25 to 30 g; equal numbers of males and females) were randomly divided into two control groups (positive and negative) and three test groups. The test groups were injected with low, middle, and high doses (2.04, 10.2, and 51 mg/kg BW, respectively) of C-dots for the bone marrow micronucleus test. Mice in the negative control group were exposed to deionized water via a single tail vein injection. Mice in the positive control group were treated with 40 mg/kg BW Cytoxan by intraperitoneal injection in the 30-h administration method. The sternum of each mouse was excised and prepared for sectioning. The bone marrow micronucleus and sperm morphology were observed under an oil lens using an Olympus microscope (Olympus Corporation, Tokyo, Japan). The results were statistically evaluated using the chi-square test with significance at *P* < 0.01.

#### S. typhimurium mutagenicity (Ames) test

The extracts and controls were added to nutrient media inoculated with *S. typhimurium* (TA97, TA98, TA100, and TA102) with or without the S9 system (*in vitro* metabolic activation system using S9 mixture). The number of colonies in each culture dish was scored after 48 h of cell culture. The plates were divided into four groups: negative, positive, positive solvent, and test groups. The test group was added to C-dot media with final doses of 0.0125, 0.025, 0.05, and 0.1 mg/plate.

## Discussion

### Characteristics of the C-dots

**Figure 1:**
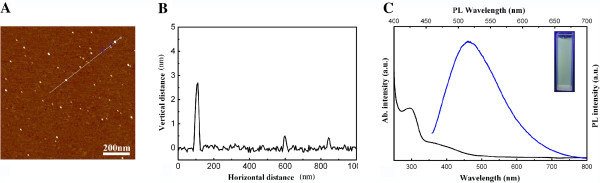
**Image, analysis, and spectra of C-dots-NH**_**2**_**.** (**A**) TM-AFM image of C-dots-NH_2_. (**B**) The section analysis selected the site in (**A**) labeled with a white line. (**C**) UV absorption and photoluminesecence spectra of C-dots-NH_2_ in pure water, the inset of the photography excited at 302 nm with an 8-W UV light.

The morphology and sectional analyses of C-dots-NH_2_ were performed by TM-AFM, and the results are shown in Figure 1A,B, respectively. The C-dots were quasispherical and uniform, with diameters ranging from 1 to 3 nm. After grafting with PEG2000N, the nanoparticle sizes slightly increased to 3 to 5 nm. The UV–vis absorption and fluorescence emission spectra of C-dots-NH_2_ are shown in Figure 1C. The peak and edge of the UV–vis spectra were at 320 and 450 nm, respectively. At an excitation wavelength of 370 nm, a strong emission peak at 540 nm was observed in the photoluminescence emission spectrum of C-dots-NH_2_. In addition, we also added the (a) statistical sizes of C-dots and C-dots-NH_2_ and (b) Zata potential (see Additional file 1: Figure S1).

### Acute toxicity evaluations

C-dot doses of 5.1 or 51 mg/kg BW did not cause mortality in the exposed mice, and no obvious clinical toxicity sign was observed. The female BALB/c mice treated with C-dots appeared healthy, and their body weight gain patterns were similar to those of the controls (*P* > 0.05) 3, 7, and 14 days after exposure. The male BALB/c mice treated with a high dose of the C-dots showed a significant difference from the control group 14 days after exposure. No significant difference was observed 3 and 7 days after exposure (*P* > 0.05), as shown in Table 1.

**Table 1:** Body weight of mouse treated with different doses of carbon dots

**Days**	**Dose**	**Female (*n*** **= 5)**	**Male (*n*** **= 5)**	**Total (*n*** **= 10)**
Day 0-1	Control	18.8 ± 0.8	18.6 ± 0.5	18.7 ± 0.7
	Low	18.0 ± 0.7	18.1 ± 0.7	18.0 ± 0.6
	High	18.6 ± 0.4	18.4 ± 0.5	18.5 ± 0.5
Day 3	Control	17.6 ± 0.4	20.3 ± 0.8	19.0 ± 1.6
	Low	18.7 ± 1.2	19.7 ± 0.8	19.2 ± 1.1
	High	19.0 ± 1.0	20.4 ± 0.9	19.7 ± 1.2
Day 7	Control	23.0 ± 0.9	21.0 ± 1.2	22.0 ± 1.5
	Low	22.6 ± 0.8	20.5 ± 1.0	21.6 ± 1.4
	High	23.3 ± 1.2	20.7 ± 0.8	22.0 ± 1.7
Day 14	Control	27.0 ± 1.1	24.7 ± 0.8	25.8 ± 1.5
	Low	27.1 ± 1.4	24.1 ± 1.2	25.6 ± 2.0
	High	25.6 ± 1.3	22.7 ± 0.6*	24.2 ± 1.8

After treatment with carbon dots, the body weight of the mouse was weighed at different time points after the administration. Data were mean ± SD. *P < 0.05 compared with control by one-way ANOVA test.

On the third day after exposure, no significant difference was found among all groups in terms of the glutamic oxaloacetic transaminase (GOT), glutamate pyruvate transaminase (GPT), urea, cholesterol, triacylglyceride (TG), blood glucose, total protein, and albumin levels (*P* > 0.05). In contrast, the creatinine (Cr) levels in the high-dose group showed significant differences (*P* < 0.01), as shown in Table 2.

**Table 2:** Biochemistry results of mice intravenously exposed to C-dots (day 3)

**Biochemical index**	**Control (*n*** **= 10)**	**Low (*n*** **= 10)**	**High (*n*** **= 10)**	***F *value**	***P *value**
Glutamate-pyruvate transaminase (U/L)	40 ± 8	45 ± 15	43 ± 7	0.597	0.558
Glutamic oxaloacetic transaminase (U/L)	108 ± 22	111 ± 31	99 ± 15	0.697	0.507
Urea (mmol/L)	8.08 ± 1.79	6.79 ± 1.10	7.13 ± 2.08	1.521	0.237
Creatinine (μmol/L)	30 ± 2	28 ± 3	26 ± 2**	9.367	0.001
Cholesterol (mmol/L)	2.82 ± 0.25	2.68 ± 0.30	2.80 ± 0.50	0.428	0.656
Triglyceride (mmol/L)	1.39 ± 0.68	1.62 ± 0.56	1.44 ± 0.43	0.468	0.632
Blood glucose (mmol/L)	8.40 ± 1.38	8.17 ± 1.08	7.50 ± 0.80	1.749	0.193
Total protein (g/L)	52.8 ± 4.0	50.8 ± 2.6	51.0 ± 2.4	1.381	0.268
Albumin (g/L)	33.3 ± 3.0	32.0 ± 2.0	31.9 ± 2.2	1.147	0.333

The biochemical parameters of mice were determined 3 days after C-dot treatment. Data were mean ± SD. **P < 0.01 compared with that from mice in the control group by one-way ANOVA test.

On the 14th day after exposure, no significant difference was found among all groups in their levels of GOT, GPT, urea, Cr, cholesterol, TG, total protein, and albumin (*P* > 0.05). Blood glucose showed significant differences from the low-dose (*P* < 0.01) and high-dose (*P* < 0.05) groups compared with the control group (Table 3). The significant decrease in the blood glucose concentration may be associated with the long duration of anesthesia.

**Table 3:** Biochemistry results of mice intravenously exposed to C-dots (day 14)

**Biochemical index**	**Control (*n*** **= 10)**	**Low (*n*** **= 10)**	**High (*n*** **= 10)**	***F *value**	***P *value**
Glutamate-pyruvate transaminase (U/L)	39 ± 11	41 ± 8	38 ± 8	0.352	0.707
Glutamic oxaloacetic transaminase (U/L)	104 ± 26	104 ± 20	94 ± 16	0.717	0.497
Urea (mmol/L)	7.66 ± 1.02	6.81 ± 1.25	6.87 ± 0.83	2.035	0.150
Creatinine (μmol/L)	24 ± 4	24 ± 3	23 ± 3	0.279	0.759
Cholesterol (mmol/L)	2.65 ± 0.50	2.67 ± 0.45	2.72 ± 0.48	0.050	0.951
Triglyceride (mmol/L)	1.66 ± 0.63	1.51 ± 0.29	1.66 ± 0.30	0.390	0.681
Blood glucose (mmol/L)	9.45 ± 1.33	7.76 ± 0.72**	8.34 ± 0.99*	6.795	0.004
Total protein (g/L)	52.2 ± 2.6	52.9 ± 2.0	52.4 ± 1.6	0.289	0.751
Albumin (g/L)	32.6 ± 0.9	32.6 ± 1.2	32.1 ± 1.2	0.825	0.449

The biochemical parameters of mice were determined at 14 days after C-dot treatment. Data were mean ± SD. *P < 0.05, **P < 0.01 compared with that from mice in the control group by one-way ANOVA test.

### Subacute toxicity evaluations

Beginning on the third week of exposure to C-dots, the body weight of the rats in all groups significantly increased (Table 4). The difference in the body weight changes of the rats between the negative groups every week was insignificant (*P* > 0.05). The food intake and food utilization of the test groups were not significantly different between the negative groups (*P* > 0.05).

**Table 4:** Diversification of rat body weight

**Gender**	**Dose**	**Number of rats**	**Initial weight**			**First week (g)**	**Second week (g)**	**Third week (g)**	**Fourth week**		
			**(g)**	***F***	***P***				**(g)**	***F***	***P***
Female	Negative control	8	193.9 ± 8.24	0.327	0.806	204.5 ± 9.4	222.6 ± 11.6	237.4 ± 16.3	246.9 ± 18.8	0.177	0.911
	Low	8	191.2 ± 7.70			201.8 ± 9.0	220.0 ± 12.1	237.4 ± 13.4	247.5 ± 12.4		
	Middle	8	194.4 ± 7.01			203.4 ± 6.8	219.9 ± 11.0	234.8 ± 13.0	246.0 ± 14.3		
	High	8	194.6 ± 7.71			204.1 ± 10.4	220.2 ± 14.1	231.9 ± 18.7	241.9 ± 21.2		
Male	Negative control	8	207.9 ± 7.9	0.970	0.421	250.8 ± 9.6	308.4 ± 13.7	344.6 ± 18.4	383.8 ± 25.5	0.590	0.626
	Low	8	210.2 ± 7.3			246.5 ± 7.7	302.1 ± 12.1	336.4 ± 7.7	373.0 ± 17.4		
	Middle	8	211.4 ± 8.8			245.9 ± 14.3	297.5 ± 16.8	336.0 ± 19.1	373.9 ± 26.2		
	High	8	205.0 ± 8.4			245.4 ± 11.4	308.5 ± 11.6	346.4 ± 15.6	383.6 ± 16.3		

Body weight of rats was taken at different time points after C-dot treatment. Data were mean ± SD. Significant difference was analyzed by one-way ANOVA test.

**Figure 2:**
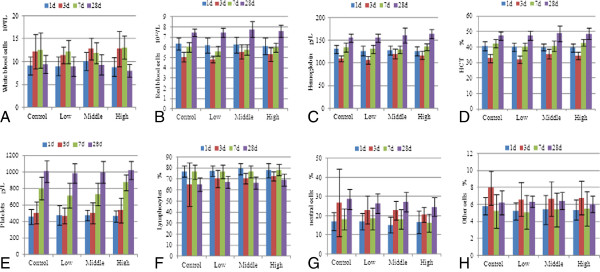
**Blood hematology analysis of rats treated with C-dots.** The rats were treated with C-dots at doses of 0.2, 2, and 20 mg/kg BW in 1, 3, 7 and 28 days. (**A**) White blood cells, (**B**) red blood cells, (**C**) hemoglobin, (**D**) HCT, (**E**) platelets, (**F**) lymphocytes, (**G**) neutral cells, and (**H**) other cells.

To reveal any potential toxic effect of the C-dots on the treated rats, biochemical and hematological analyses were performed. The following key hematology markers were assessed at various time points (1, 3, 7, and 28 days): white blood cells, red blood cells, platelets, lymphocytes, neutral cells, other cells, hemoglobin, and hematocrit (HCT) (Figure 2). All above parameters in rats treated with different concentrations of C-dots at different time points appeared to be normal compared with the control groups. However, 7 days after exposure, the HCT of the low-dose C-dot-treated group showed a significant difference compared with that of the normal control group (*P* < 0.05).

**Figure 3:**
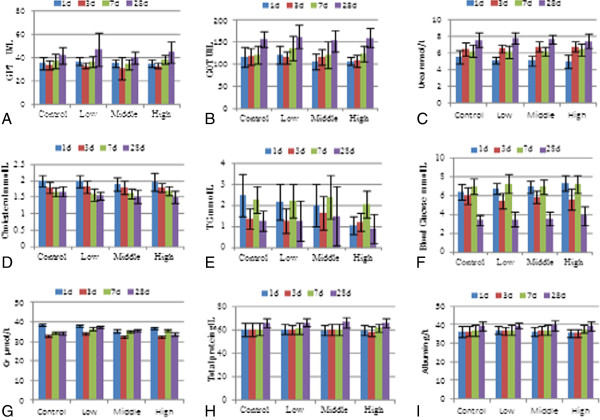
**Changes of the blood biochemical data of rats treated with C-dots.** The rats were treated with C-dots at doses of 0.2, 2, and 20 mg/kg BW in 1, 3, 7, and 28 days. (**A**) GPT, (**B**) GOT, (**C**) urea, (**D**) cholesterol, (**E**) TG, (**F**) blood glucose, (**G**) Cr, (**H**) total protein, and (**I**) albumin.

Subacute C-dot poisoning can cause changes in the following biochemical indices: GOT, GPT, urea, Cr, cholesterol, TG, blood glucose, total protein, and albumin (Figure 3). On the first day after exposure, the blood arsenic level in the high-dose group was obviously higher than in the control group (*P* < 0.01), and the TG levels significantly decreased (*P* < 0.01). Within 30 days after the first exposure, all biochemical parameters of the rats treated with different doses of C-dots at different time points appeared to be normal compared with the control groups. Although some of the biochemical and hematological parameters were statistically different between the test and negative control group, these differences were not biologically significant.

**Figure 4:**
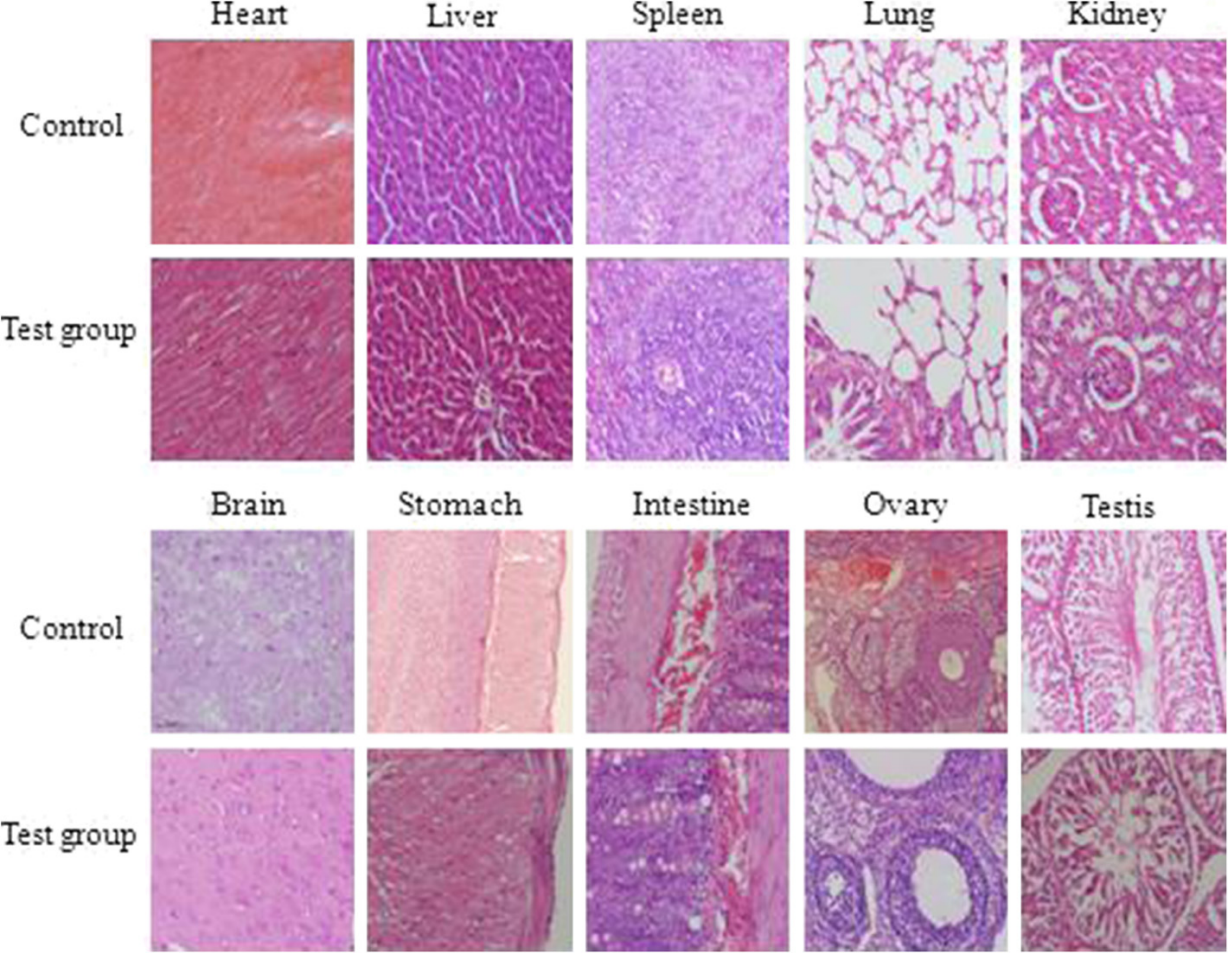
**Results of histopathological analyses of rats.** The rats were treated with C-dots at the dose of 20 mg/kg BW at 30 days.

The organs of the rats injected with C-dots at the highest dose of 20 mg/kg BW were harvested for histopathological analysis. These organs included the heart, liver, spleen, stomach, kidneys, lungs, brain, stomach, intestines, ovaries, and testes. As shown in Figure 4, no obvious organ damage was noticed. Likewise, no apparent histopathological abnormality or lesion in the test groups was observed. The size and structure of the cardiac muscle fibers in the test group were uniform and normal. There was no steatosis, necrosis, or hydropic degeneration in the exposed hepatic sections. The structure of the liver lobule was normal, with few collagen fibers located in the portal area and central vein. The splenic capsule was complete, and the red and white pulps were clear. The lung structures were normal and no inflammation was found. In the sections of the kidneys, the glomerular structure was easily distinguished. No bleeding, ulcer, or abnormal differentiation was observed in the gastric mucosa.

No significant difference was found between the weights of the major organs (liver, spleen, kidney, ovaries, and testes) between the test groups (both female and male) and negative control group (*P* > 0.05), as shown in Table 5.

**Table 5:** Diversification of rat major organ weight

**Gender**	**Dose**	**Body weight (g)**	**Liver**		**Spleen**		**Kidney**		**Ovarian/testis**	
			**Wet weight (g)**	**Liver/body (%)**	**Wet weight (g)**	**Spleen/body (%)**	**Wet weight (g)**	**Kidney/body (%)**	**Wet weight (g)**	**Organs/body (%)**
Female	Negative control	235.9 ± 17.2	6.74 ± 0.66	2.86 ± 0.22	0.60 ± 0.07	0.26 ± 0.03	1.78 ± 0.14	0.75 ± 0.05	0.24 ± 0.07	0.10 ± 0.03
	Low	235.8 ± 12.8	6.92 ± 0.53	2.94 ± 0.20	0.59 ± 0.04	0.25 ± 0.02	1.70 ± 0.12	0.72 ± 0.03	0.27 ± 0.05	0.12 ± 0.02
	Middle	234.9 ± 13.9	6.61 ± 0.53	2.83 ± 0.30	0.59 ± 0.03	0.25 ± 0.01	1.71 ± 0.09	0.73 ± 0.05	0.25 ± 0.05	0.11 ± 0.02
	High	230.8 ± 20.6	6.67 ± 0.90	2.88 ± 0.22	0.56 ± 0.07	0.24 ± 0.02	1.76 ± 0.12	0.76 ± 0.03	0.26 ± 0.06	0.11 ± 0.02
Male	Negative control	362.5 ± 22.7	12.52 ± 1.94	3.44 ± 0.34	0.91 ± 0.14	0.25 ± 0.04	2.79 ± 0.25	0.77 ± 0.05	3.13 ± 0.13	0.86 ± 0.03
	Low	352.9 ± 17.8	11.21 ± 1.05	3.18 ± 0.24	0.90 ± 0.08	0.25 ± 0.02	2.65 ± 0.23	0.75 ± 0.07	3.19 ± 0.16	0.90 ± 0.06
	Middle	354.1 ± 27.0	11.22 ± 1.02	3.18 ± 0.30	0.86 ± 0.10	0.24 ± 0.03	2.61 ± 0.16	0.74 ± 0.05	3.21 ± 0.18	0.91 ± 0.04
	High	362.1 ± 15.3	11.16 ± 0.91	3.08 ± 0.26	0.90 ± 0.72	0.25 ± 0.02	2.66 ± 0.16	0.73 ± 0.04	3.21 ± 0.19	0.89 ± 0.05

The liver, spleen, kidney, and ovary/testis of rats were separated and weighed. Data were mean ± SD. Significant difference was analyzed by one-way ANOVA test.

### Medullary micronucleus test

Table 6 shows that the micronucleus cell frequency (MCF) of hematopoietic cells in the mouse bone marrow and the polychromatic erythrocyte/normochromatic erythrocyte (PCE/NCE) were all within the normal range. The MCF results of the positive group were higher than that of the negative group (*P* < 0.01). All C-dot dosages did not induce micronucleus formation in the mouse cells.

**Table 6:** Medullary micronucleus results of mice exposed to C-dots

**Gender**	**Dose**	**No.**	**PCE**	**Micronucleus PCE**		**Micronucleus cell rate ( ‰)**	***P *value**	**PCE/NCE**
				**No.**	$\bar{X}$ **± *S***			
Female	Negative control	5	5 × 1,000	5	1.0 ± 0.7	1.0		1.33 ± 0.18
	Low	5	5 × 1,000	4	0.8 ± 0.8	0.8		1.33 ± 0.31
	Middle	5	5 × 1,000	4	0.8 ± 0.4	0.8		1.33 ± 0.19
	High	5	5 × 1,000	5	1.0 ± 0.7	1.0		1.28 ± 0.19
	Positive control	5	5 × 1,000	157	31.4 ± 5.8***	31.4	0.000	1.23 ± 0.08
Male	Negative control	5	5 × 1,000	2	0.4 ± 0.5	0.4		1.41 ± 0.12
	Low	5	5 × 1,000	3	0.6 ± 0.5	0.6		1.40 ± 0.08
	Middle	5	5 × 1,000	2	0.4 ± 0.5	0.4		1.36 ± 0.11
	High	5	5 × 1,000	3	0.6 ± 0.5	0.6		1.41 ± 0.10
	Positive control	5	5 × 1,000	163	32.6 ± 6.4***	32.6	0.000	1.22 ± 0.07

Data were mean ± SD. ***P < 0.001 compared with that from the negative control. Significant difference was analyzed by the chi-square test.

#### *S. typhimurium* mutagenicity (Ames) test

The results of the Ames test showed that no detectable mutagenicity was caused by the C-dots under the experimental conditions, as shown in Table 7. Strains TA97, TA98, and TA102 were induced by dexon (50 μg/plate), whereas strain TA100 was treated with sodium azide (1.5 μg/plate) without the addition of the S-9 system. Strains TA97, TA98, and TA100 were induced by 2-acetylaminofluroene (5 μg/plate), whereas strain TA102 was treated with 8-dihydroxy-anthraquinone (50 μg/plate) when the S-9 system was added. Except for TA100, all strains were induced positively by the solvent dimethylsulfoxide with the S-9 system added.

**Table 7:** Ames test results of mice (revertant colonies)

**Dose (mg/plate)**		**Strains**			
		**TA97**	**TA98**	**TA100**	**TA102**
0.1	-S9	129.3 ± 11.4	32.3 ± 6.7	134.7 ± 20.0	290.0 ± 33.4
	+S9	128.7 ± 25.0	38.0 ± 6.9	138.3 ± 13.2	294.0 ± 28.0
0.05	-S9	128.7 ± 15.1	33.0 ± 7.8	132.0 ± 16.0	279.3 ± 22.0
	+S9	139.3 ± 8.3	35.7 ± 5.5	132.0 ± 18.3	295.7 ± 14.4
0.025	-S9	131.3 ± 9.0	33.0 ± 7.2	128.7 ± 12.2	280.0 ± 13.1
	+S9	142.0 ± 11.1	40.0 ± 5.3	151.0 ± 13.5	302.3 ± 19.3
0.0125	-S9	118.0 ± 13.5	33.3 ± 6.4	127.7 ± 19.7	279.3 ± 28.4
	+S9	121.3 ± 11.0	34.0 ± 6.5	134.7 ± 16.2	284.3 ± 17.6
Negative control	-S9	135.3 ± 14.7	31.0 ± 4.6	136.7 ± 24.4	294.0 ± 27.5
	+S9	131.0 ± 26.5	41.0 ± 4.0	130.7 ± 18.0	288.7 ± 20.4
Positive solvent group	-S9	130.3 ± 14.6	33.7 ± 4.2	-	284.0 ± 20.3
	+S9	130.7 ± 12.1	34.7 ± 6.1	137.3 ± 13.3	295.3 ± 21.4
Positive control	-S9	803.3 ± 165.0	893.3 ± 220.3	640.0 ± 91.7	946.7 ± 122.2
	+S9	780.0 ± 177.8	1,160.0 ± 183.3	746.7 ± 140.5	1,000.0 ± 208.8

The number of colonies in each culture dish was scored after 48 h of cell culture. Data were mean ± SD.

## Conclusion

In this work, photoluminescent C-dots with good stability, water solubility, and high dispersibility were successfully prepared. The toxicity of the prepared C-dots was then systematically evaluated. The results showed that the fluorescent C-dots at difference doses did not exert any significant toxic effect on rats and mice under the doses used in our experiments. No abnormality or lesion was observed in the major organs of rats treated with the C-dots. The C-dots also did not exhibit any gene toxicity. Thus, the as-prepared C-dots have good biocompatibility and potential use in *in vivo* molecular imaging and biolabeling, and others.

## Competing interests

The authors declare that they have no competing interests.

## Authors’ contributions

KW and ZG participated in the animal experiment. GG, YW, and YW designed and participated in the animal experiments. GS synthesized the photoluminescent carbon dots evaluated in this research. DC participated in the design and the coordination of this study. All authors read and approved the final manuscript.

## Supplementary Material

Additional file 1: Supplementary data A document showing the preparation/production of C-dots. Click here for file
