# Supplementary material for: Systematic safety evaluation on photoluminescent carbon dots
Source: Nanoscale Res Lett. 2013 Mar 8;8(1):122. doi: 10.1186/1556-276X-8-122 (PMC3602655; doi:10.1186/1556-276X-8-122)
Supplement: Additional file 1: Supplementary data — A document showing the preparation/production of C-dots. [file 1556-276X-8-122-S1.doc]

**Supplementary data:**

C-dots are one typical kind of carbon nanomaterials. In our experiments, we prepared the C-dots is spherical with range of 3-5nm in diameter using nitric acid oxidation method using the commercial raw soot. Here, the nitric acid oxidation pays a key role to generate many carboxyl groups and epoxy groups on the surface of C-dots, just like chemical preparation of the Graphene oxides us ing the Graphite powder, thus imparting them with excellent water solubility and the suitability for subsequent functionalization with various organic, polymeric, inorganic, or biologica species. Using the technology, we can realize to produce a large-scale C-dots once time, which also can ensure provide the same batch samples to conduct thereafter series of safety evaluation experiments.

In addition, we also added the statistical sizes of C-dots Figure 1s( a) and C-dots-NH_2_ and Zata potential Figure 1s(b). The size distribution was gained by analyzing more than ten pieces of AFM images. The diameters of C-dots and C-dots-NH_2_ are average 2.8 ± 0.5 nm and the 5.9± 0.35 nm, respectively. Their Zata potentials are ~-23.8 and ~ -8.3mV.


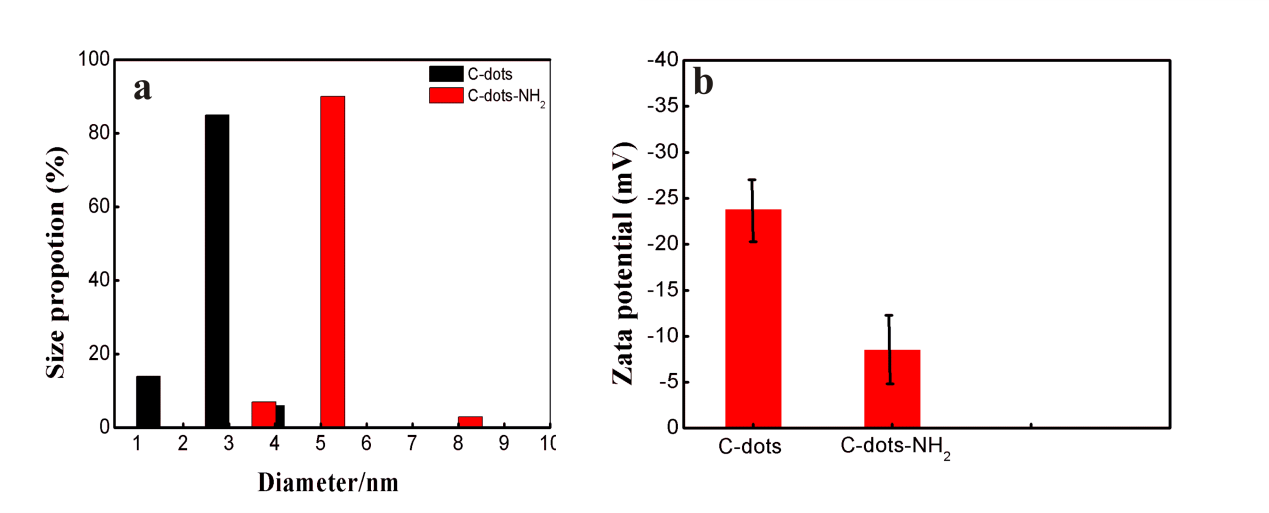


Figure 1s a) the size distribution of the C-dots and C-dots-NH_2_; b) Zata potentials of C-dots and C-dots-NH_2_
